# Optimization of Pyocyanin Production by *Pseudomonas aeruginosa* OG1 Using RSM: In Vitro Evaluation of Its Antibacterial and Anticandidal Efficacy Against Some Pathogens

**DOI:** 10.3390/antibiotics15040330

**Published:** 2026-03-25

**Authors:** Levent Dikbaş, Şeyma Alım, Sevda Uçar, Murat Özdal, Neslihan Dikbaş

**Affiliations:** 1 Turkey In Vitro Fertilization Center, Qarshi 180100, Uzbekistan; ldikbas@hotmail.com; 2 Department of Agricultural Biotechnology, Faculty of Agriculture, Ataturk University, Erzurum 25000, Turkey; seyma.alim14@ogr.atauni.edu.tr; 3 Department of Field Crops, Faculty of Agricultural Sciences and Technology, Sivas Science and Technology University, Sivas 58000, Turkey; sucar@sivas.edu.tr; 4 Department of Biology, Science Faculty, Ataturk University, Erzurum 25000, Turkey; mozdal@atauni.edu.tr

**Keywords:** *Pseudomonas aeruginosa*, pyocyanin, response surface methodology, antimicrobial activity, optimization

## Abstract

**Background:** The increasing antimicrobial resistance has led to a greater demand for alternative treatment options, which in turn has increased interest in naturally occurring biomolecules such as pyocyanin. **Methods:** In this study, a three-factor Box–Behnken Design (BBD)-based response surface methodology (RSM) was employed to optimize the effects of glycerol, peptone, and pH on pyocyanin production by *Pseudomonas aeruginosa* OG1. The antimicrobial efficacy of the optimized pyocyanin was subsequently evaluated in vitro against three Candida species and four clinically important bacterial pathogens using the disk diffusion method, with gentamicin and fluconazole used as positive controls. **Results:** The second-order polynomial model demonstrated excellent fit (F = 176.3, *p* < 0.0001) with a non-significant lack of fit, indicating adequate representation of the experimental data. The optimal conditions were determined to be glycerol at 1.11% (*w*/*v*), peptone at 17.86 g/L, and a pH of 7.27, yielding a predicted pyocyanin concentration of 25.92 mg/L. Antimicrobial testing revealed broad-spectrum, dose-dependent activity against all tested microorganisms. The highest efficacy was observed against *Bacillus cereus* (26.4 ± 1.3 mm at 40 µg/mL), followed by *Candida glabrata* (21.5 ± 1.6 mm), *Klebsiella pneumoniae* (17.6 ± 1.4 mm), *Candida albicans* (15.4 ± 1.8 mm), *Candida parapsilosis* (13.2 ± 1.9 mm), *Proteus mirabilis* (12.5 ± 1.3 mm), and MRSA *Staphylococcus aureus* (9.2 ± 1.1 mm). **Conclusions:** These findings demonstrate that BBD-based RSM is a robust approach for optimizing pyocyanin production and that pyocyanin represents a promising dose-dependent antimicrobial agent against susceptible pathogens.

## 1. Introduction

Pigments have been widely used throughout history as coloring agents in food, textiles, plastics, cosmetics, pharmaceuticals, and other industrial fields, and, today, with the advancement of technology, they have become indispensable components of industrial production [1,2]. Synthetic pigments have been preferred on an industrial scale for many years due to their low cost, wide color range, and high stability [3]. However, despite these advantages, the toxic, carcinogenic, and poorly biodegradable nature of synthetic pigments has led researchers to seek natural pigments, which are safer, more sustainable, and environmentally friendly [4,5].

Natural pigments are secondary metabolites synthesized by plants and microorganisms; they are environmentally friendly, non-toxic, and biodegradable and exhibit various biological activities such as antimicrobial, antioxidant, and antiviral properties [6,7]. Among these natural pigments, those of microbial origin offer significant advantages over plant pigments because they can be produced quickly and in high quantities in low-cost culture media, demonstrate biological activity, can be obtained in high yield, and possess wide chemical diversity; therefore, they are attracting more attention in terms of industrial applicability [7,8,9].

Microorganisms produce natural pigments such as pyocyanin, carotenoid, quinone, riboflavin, prodigiosin, indigo, and melanin, and they can be used to support human health and environmental sustainability [1,10,11]. Among these pigments, pyocyanin, produced as a secondary metabolite by *P. aeruginosa* strains, is a water-soluble, blue-green, phenazine derivative extracellular pigment [12,13]. Pyocyanin possesses various biological activities, including antiparasitic, immunosuppressive, anticancer, antimicrobial, antimalarial, antioxidant, anticandidal, and biofilm-resistant properties [4,14]. The effect of this secondary metabolite, which has a phenazine structure, particularly on fungal pathogens, has become an increasingly researched topic in recent years [12,15,16]. *Candida* species are among the major fungal pathogens that tend to develop resistance to antifungal agents [17]. Pyocyanin exerts a fungicidal effect by disrupting the cell membrane integrity of various *Candida* species [18,19]. Because of these properties, pyocyanin is becoming a potential candidate for use in various industrial fields such as food, biotechnology, medicine, pharmaceuticals, textiles, biological control, engineering, and nanotechnology [18,20].

With the development of industry and the increase in biotechnological applications, how to achieve more economical and sustainable production of biological pigments has become an important research topic in the field of microbial biotechnology [21]. The production of pyocyanin, an industrially important secondary metabolite, varies considerably depending on the physiological state of the microorganism, as well as environmental and nutritional factors [22]. Therefore, to make pigment production more economical and efficient, it is necessary to optimize metabolite synthesis in addition to microbial growth [23].

Traditional univariate optimization methods are time-consuming and do not provide sufficiently accurate results for bioprocesses because they only evaluate the effect of a single parameter [24,25]. To overcome these drawbacks, statistical modeling techniques that evaluate multiple variables simultaneously are coming to the forefront. The preferred RSM (response surface methodology) in the present study is an empirical model that involves the use of statistical and mathematical methods to correlate multiple important input variables (pH, carbon, nitrogen source, etc.) with the response [26]. Because it considers the interactions between variables, obtains the maximum amount of data with a minimum number of experiments, saves time, and provides more accurate results, it is highly preferred in bioprocess engineering [27,28].

In the present study, the effect of environmental conditions (glycerol, peptone, and pH) on pyocyanin production by *P. aeruginosa* was optimized using RSM, and the antimicrobial efficacy of the produced pigment against some clinically important pathogens was evaluated in vitro.

## 2. Results and Discussion

RSM is a mathematical and statistical method successfully used to optimize the environmental components of any fermentation process [29]. The main objective of this study is to optimize the production of pyocyanin, an important virulence factor and redox-active phenazine pigment produced by *P. aeruginosa*, by systematically investigating the effects of three critical environmental components, and to evaluate the antimicrobial efficacy of the produced pyocyanin against some pathogens. In this study, a total of 15 experiments were conducted using the Box–Behnken Design (BBD), with three variables and three different levels of these three variables. The experimental responses of the variables are presented in Table 1.

RSM was successfully applied to optimize pyocyanin production in *P. aeruginosa* by varying glycerol, peptone, and pH. The second-order polynomial regression model equation describing the relationship between the response (pyocyanin) and the independent variables (glycerol (A), peptone (B), and pH (C)) is given below:

**Pyocyanin (mg/L) =** −263.3 + 55.43[A] + 2.928[B] + 63.89[C] − 30.90[A^2^] −

0.10[B^2^] − 4.575[C^2^] + 0.144[A × B] + 1.40[A × C] + 0.066[B × C]                 

The second-order polynomial model developed for pyocyanin production was statistically evaluated using ANOVA (Table 2). The model statistics for this zero-order correlation yielded an F-value of 176.3 with a *p*-value < 0.0001, indicating statistical significance. The R^2^ (0.9969) values showed that 99.69% of the variability was explained by the model, revealing an excellent correlation between the predicted and experimental pyocyanin yield values. All linear terms were significant at the *p* < 0.0001 level, including glycerol (F = 304.7), peptone (F = 376.5), and pH (F = 273.4). All quadratic terms were similarly highly significant (*p* < 0.0001), with glycerol^2^ (F = 197.9) being the strongest factor, followed by peptone^2^ (F = 221.5) and pH^2^ (F = 181), suggesting a curvilinear parameter–pyocyanin yield relationship in each case. All three two-way interaction terms—glycerol × peptone (*p* = 0.016), glycerol × pH (*p* = 0.031), and peptone × pH (*p* = 0.024)—were statistically significant at the α = 0.05 level. The non-significant lack of fit indicated that the model described the experimental data sufficiently well over the range of study.

The regression results (Table 3) showed that glycerol had a significant positive linear effect on pyocyanin production. This observation is consistent with earlier research indicating that glycerol is an effective carbon source for *P. aeruginosa* and produces higher pyocyanin yields than other carbon substrates [18,22]. However, the strong negative quadratic coefficient for glycerol indicates that increasing the glycerol concentration improves pyocyanin production only up to an ideal level, after which additional increases result in a decrease in yield. Peptone was also found to be a highly significant factor in pyocyanin synthesis. Peptone, a rich source of amino acids and peptides, can provide essential precursors for the manufacture of pyocyanin and other phenazine pigments [6]. Increasing the peptone concentration from 8 g/L to 16 g/L significantly increased pigment production, corroborating the observation that nitrogen-rich environments promote phenazine biosynthesis. The impact of pH was similarly considerable. Both acidic and severely alkaline environments inhibited pyocyanin production, but slightly alkaline circumstances promoted pigment synthesis. This finding is consistent with that of prior research demonstrating that moderately alkaline circumstances are favorable for pyocyanin synthesis in *P. aeruginosa* [9,18].

**Figure 1:**
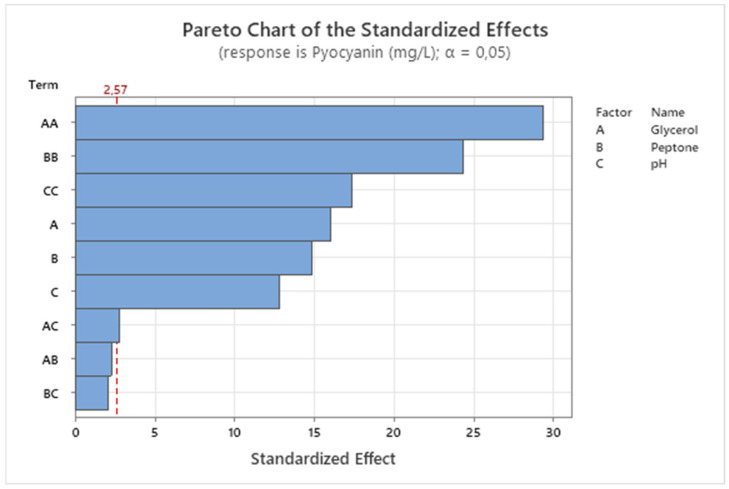
A Pareto chart for pyocyanin production.

A pareto chart was prepared to show the relative effects of the factors on the response and is presented in Figure 1. The graph shows that the squared term (AA) of glycerol was the most dominant and significant factor influencing the response, supporting the ANOVA results. Similarly, it was observed that the squared terms of peptone and pH (BB > CC) had significant effects on pyocyanin production, while other factors (A > B > C > AC) had relatively less significant effects. All pairwise interaction terms exceeded the significance threshold in the Pareto chart, consistent with the ANOVA results showing significant two-way interactions (AB: *p* = 0.016; AC: *p* = 0.031; BC: *p* = 0.024).

**Figure 2:**
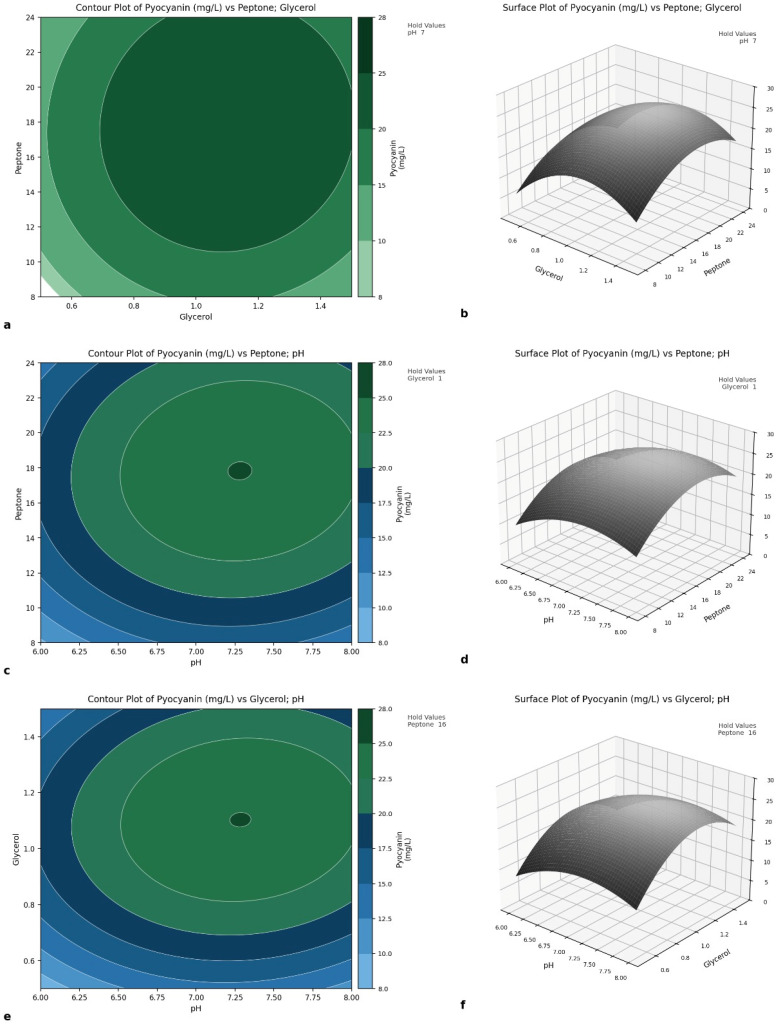
Surface plot for pyocyanin production: effect of peptone and glycerol (**a**,**b**), effect of peptone and pH (**c**,**d**), effect of glycerol and pH (**e**,**f**).

Three-dimensional surface (and contour) plots visually reveal the combined effect of two factors on the response while keeping one factor at a moderate level constant. Three-dimensional response surface (Figure 2a) and contour (Figure 2b) plots were constructed to illustrate the combined effects of glycerol and peptone concentrations on pyocyanin production by *P. aeruginosa* OG1 at optimal pH (7.27). Glycerol and peptone individually had a significant effect on production, but the interaction between glycerol and peptone was not significant. Pyocyanin production initially increased with increasing amounts of glycerol and peptone, but after a certain level, increases in both factors decreased production. The interaction between peptone and pH is presented in Figure 2c,d. Although peptone and pH individually exerted significant effects on pyocyanin production, their binary interaction (BC) was also statistically significant (F = 10.02, *p* = 0.024), suggesting that the effect of pH on pigment biosynthesis is partially dependent on the available nitrogen concentration. Pyocyanin production increased in parallel with increasing peptone and pH, but a decrease was observed at higher peptone and pH values. The effects of glycerol and pH on pyocyanin production are presented in Figure 2e,f, and it was observed that the binary interaction of these factors is significant. Pyocyanin production increased with increasing glycerol and pH but began to decrease after a certain point. Specifically, when glycerol and pH were simultaneously low (0.5% and pH 6), pyocyanin production decreased more sharply than predicted by univariate effects, indicating synergy between the two parameters. These findings confirm that pyocyanin production varies depending on nitrogen and carbon availability, the presence of different compounds, and various environmental factors such as temperature and pH [18,20,30].

**Figure 3:**
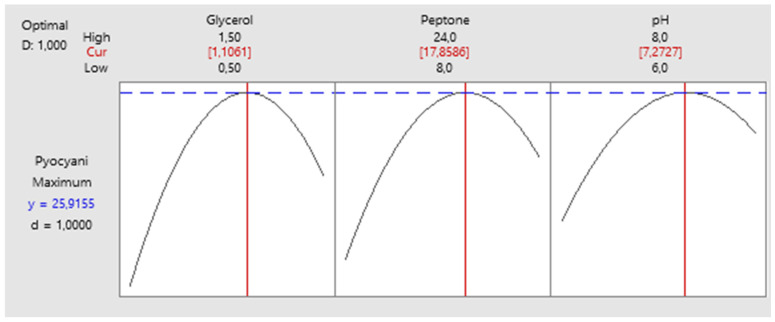
Desirability optimization plot showing predicted optimal conditions for maximum pyocyanin production by *P. aeruginosa* OG1.

**Figure 4:**
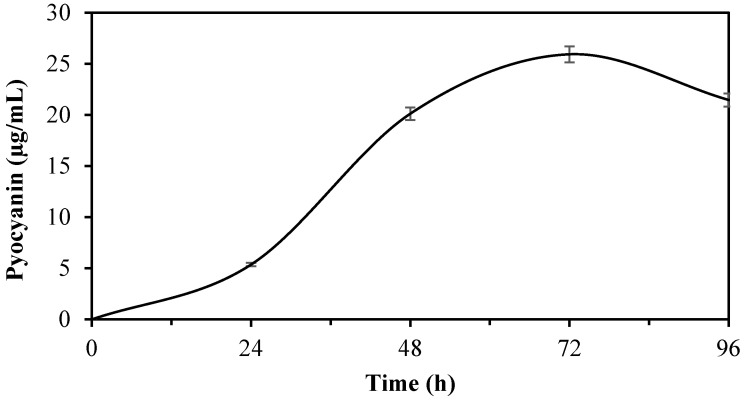
Time course of pyocyanin production under optimized conditions.

Based on the polynomial equation model, the optimal conditions for maximum pyocyanin production by *P. aeruginosa* were estimated to be glycerol at 1.11% (*w*/*v*), peptone at 17.86 g/L, and a pH of 7.27, with a predicted pyocyanin content of 25.41 mg/L under these conditions (Figure 3). The findings are consistent with those of the existing literature reporting that pyocyanin production is generally maximized under near-neutral, slightly alkaline conditions in environments rich in peptone and containing a suitable carbon source such as glycerol [31,32]. As shown in Figure 4, pyocyanin production reached a maximum of 25.41 ± 0.38 mg/L at the 72nd hour of incubation. This result is in excellent agreement with the model-predicted optimal yield of 25.92 mg/L, corresponding to a relative error of 2.0%, thereby confirming the high predictive reliability of the BBD-based RSM model.

**Figure 5:**
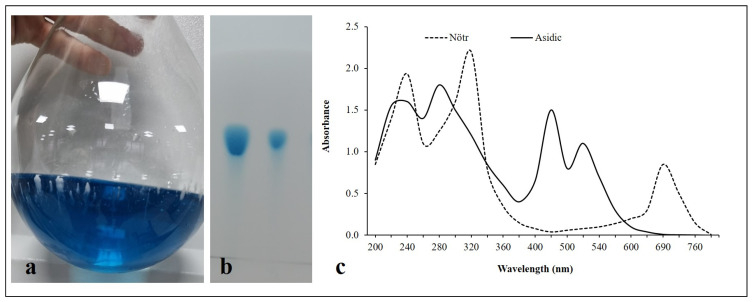
(**a**) Deep blue chloroform extract obtained from *P. aeruginosa* culture supernatant, indicating successful extraction of pyocyanin into the organic phase. (**b**) The thin-layer chromatography (TLC) profile shows the isolation of the blue pigment on a silica gel 60 F254 plate. Lane 1 (Left): Standard (Pure) pyocyanin; Lane 2 (Right): Purified pyocyanin produced in this study. (**c**) UV–visible absorption spectra of pyocyanin produced by *P. aeruginosa* under neutral (solid line) and acidic (dashed line) conditions. The spectral scan was performed between 200 and 800.

After the bacterial culture was centrifuged, the supernatant was subjected to liquid–liquid extraction using chloroform, producing a deep blue organic phase that is indicative of pyocyanin (Figure 5a). The efficient partitioning of pyocyanin into the organic phase was confirmed by the strong blue coloration of the chloroform layer, which is in line with its hydrophobic nature and solubility characteristics previously reported by Essar et al. (1990) [33] and Marey et al. (2024) [7].

Using chloroform:methanol (90:10, *v*/*v*) as the mobile phase, TLC analysis of the extracted pyocyanin on silica gel 60 F254 plates produced a single blue spot with an Rf value consistent with that reported in the literature (0.70–0.81) (Figure 5b), confirming the identity and purity of the isolated pigment [4,34]. The effective extraction of pyocyanin from the *P. aeruginosa* isolate was further confirmed by the co-migration of the extracted sample with the commercial pyocyanin standard at the same Rf value, which is consistent with the results published by Shouman et al. (2023) [35] and Marey et al. (2024) [7].

Pyocyanin’s known pH-dependent spectrum behavior was supported by its UV–vis absorption spectra in both neutral and acidic environments (Figure 5c). Consistent with previously published data [36,37], the neutral form showed distinctive absorption peaks at ~320 nm and ~690 nm, which correspond to π-π* transitions of the phenazine ring system and the blue-green coloration of *P. aeruginosa*, respectively. Shouman et al. (2023), who described pyocyanin obtained from both clinical and environmental *P. aeruginosa* isolates, further support these conclusions [35]. Protonation of the phenazine nitrogen atoms caused a bathochromic shift under acidic conditions, shifting the visible absorption band to 460–520 nm and giving the protonated form its distinctive yellow-orange color [38]. Marey et al. (2024) reported that the UV–Vis absorbance maxima at 215, 265, 385, and 520 nm for pyocyanin dissolved in 0.1 N HCl [7]. When combined, these results validate the pH-dependent optical characteristics of pyocyanin and prove the identity of the isolated pigment.

In addition to optimizing fermentation conditions, the biological potential of the pyocyanin produced in the study was investigated by evaluating its antimicrobial activity against some clinically important pathogens, including yeasts (*C. albicans*, *C. glabrata*, and *C. parapsilosis*) and bacteria (*K. pneumoniae*, *B. cereus*, *MR S. aureus*, and *P. mirabilis*) (Table 4).

Pyocyanin was tested against pathogens at three different concentrations, and the differences between these concentrations were found to be statistically significant (*p* < 0.05). The pathogen most susceptible to pyocyanin was the Gram-positive bacterium *B. cereus*, which had the largest zone diameter (26.4 mm at the highest dose), followed by C. *glabrata*, *K. pneumoniae*, *C. albicans*, *P. mirabilis*, and *C. parapsilosis*. The fact that the highest efficacy was observed against *B. cereus* is consistent with many previous studies reporting that Gram-positive bacteria are more susceptible to pyocyanin than Gram-negative bacteria [18,39,40]. This situation can be explained by differences in cell wall structure (particularly lipid content) [40]. In line with our findings, Hamad et al. (2020) obtained similar antimicrobial efficacy against *B. cereus* and *K. pneumoniae* with pyocyanin application and determined the zone regions to be in the range of 16.2–32.2 mm and 10.8–26.0 mm, respectively [16]. For many pathogens (*B. cereus*, *C. albicans*, and *P. mirabilis*), especially for *C. glabrata* and *K. pneumoniae*, the diameter of the inhibition zone increased in parallel with increasing concentration. Similarly, Oves et al. (2024) observed a 24 mm inhibition zone against *C. albicans* in the application of pyocyanin and reported that the diameter of the inhibition zone increased with concentration [15]. Additionally, Thukkaram et al. (2024) reported that pyocyanins isolated from different *P. aeruginosa* strains had a good inhibitory effect against different bacteria and *Candida* [41]. These findings support the potential of pyocyanin as a dose-dependent antimicrobial agent [16]. In contrast, *C. parapsilosis* did not show a significant difference in zone diameter among the tested doses, and the inhibition diameter decreased at the highest concentration. This suggests a potential tolerance mechanism or experimental variability. In contrast, MRSA showed the lowest susceptibility among all tested organisms (6.4 to 9.2 mm at 20–40 µg/mL), and none of the tested concentrations produced an inhibition zone comparable to that of the gentamicin-positive control. In contrast to these findings, Kamer et al. (2023) reported that pyocyanin exhibited dose-dependent antibacterial activity against MRSA and created inhibition zones with ranges of 15–36 mm, 13–30 mm, and 10–19 mm at concentrations of 40, 20, and 10 µg/mL [42]. The results obtained indicate that the antimicrobial effect of pyocyanin on MRSA may vary depending on the dose used, the strain, and the experimental conditions.

## 3. Materials and Methods

### 3.1. Bacterial Strain and Inoculum Preparation

*P. aeruginosa* OG1 was used as the pyocyanin-producing strain [4]. The culture was maintained on nutrient agar (Oxoid, Basingstoke, UK) plates and stored at 4 °C for short-term use. For all experiments, a single colony was transferred into nutrient broth and incubated overnight at 30 °C with shaking (180 rpm). The resulting culture was adjusted to a standardized optical density (OD600 = 1.0).

### 3.2. Experimental Design and Optimization Variables

The basal medium (g/L) contained Lab-Lemco 1.0, yeast extract (Merck, Darmstadt, Germany) 2.0, and NaCl ((Isolab, Eschau, Germany) 5.0. Glycerol (Isolab, Eschau, Germany) and peptone (HiMedia, Mumbai, India) were added at the specified levels. A three-factor, three-level Box–Behnken Design (BBD) was employed to optimize pyocyanin production. The independent variables investigated were glycerol concentration (0.5–1.5%, *w*/*v*), peptone concentration (8–24 g/L), and initial pH (6.0–8.0). Each factor was coded at three levels: −1 (low), 0 (center), and +1 (high). Experimental conditions were selected to cover low, medium, and high levels of each factor. Each experimental condition was prepared in 250 mL Erlenmeyer flasks containing 50 mL of production medium. The media were sterilized at 121 °C for 15 min, and pH was adjusted prior to autoclaving. Flasks were inoculated with 1% (*v*/*v*) of overnight culture. All cultures were incubated at 30 °C and 180 rpm for 72 h under aerobic conditions. At the end of incubation, the cultures were used for pyocyanin quantification.

### 3.3. Pyocyanin Quantification

To determine the amount of pyocyanin, the cell-free supernatant was obtained by centrifugation at 10,000× *g* for 10 min. Then, 2.5 mL of the supernatant was acidified with 0.5 mL of 0.2 N HCl (Merck, Darmstadt, Germany) and extracted with 1.5 mL of chloroform (Isolab, Eschau, Germany). The mixture was vortexed and centrifuged at 5000× *g* for 10 min to separate the phases. The absorbance of the chloroform layer (acidic pyocyanin) was measured at 520 nm using a UV–Vis spectrophotometer (Agilent Technologies, Santa Clara, CA, USA). Pyocyanin concentration was determined in mg/L according to the equation A_520_ × 17.072.

### 3.4. Purification and Characterization of Pyocyanin

At the end of fermentation, the culture liquid was centrifuged at 5000 rpm for 10 min to obtain a cell-free supernatant. Pyocyanin was then recovered by extensive liquid–liquid extraction using an equal volume of chloroform. The resulting sub-chloroform phase was concentrated and further purified by silica gel column chromatography (30 cm × 2 cm) using chloroform as the mobile phase. The final purification was performed via thin-layer chromatography (TLC) on silica gel 60 F254 plates (Merck, Darmstadt, Germany), as previously described by Cheluvappa (2014) [34] and Ozdal et al. (2019) [4]. A mixture of methanol (Merck, Darmstadt, Germany) and chloroform at a ratio of 90:10 *v/v* was used as the mobile phase. The purified pyocyanin was dissolved in methanol, and its UV–Vis absorption spectrum was recorded in the range of 200–800 nm.

### 3.5. Antimicrobial Activity Assay

The antimicrobial activity of the extracted pyocyanin was tested against a panel of indicator microorganisms, including yeasts (*C. albicans, C. glabrata, and C. parapsilosis*) and bacteria (*K. pneumoniae* ATCC 70063, *B. cereus* BC6830, methicillin-resistant *S. aureus* [MRSA] ATCC 67106, and *P. mirabilis* ATCC 12453). The assay was performed using the disk diffusion method [43]. The test microorganisms were grown overnight in appropriate broth media (e.g., nutrient broth for bacteria and Sabouraud dextrose broth for yeasts) and adjusted to 0.5 McFarland turbidity. Suspensions were spread uniformly onto Mueller–Hinton agar (MHA; bacteria) (Oxoid, Basingstoke, UK) or Sabouraud dextrose agar (SDA; yeasts) (HiMedia, Mumbai, India) plates. Sterile paper disks (6 mm diameter) were impregnated with 100 µL of pyocyanin solution at each concentration (20, 30, and 40 µg/mL) by applying the solution in multiple sequential aliquots with intermediate drying steps. Methanol was then completely evaporated from the impregnated disks under laminar flow conditions prior to placement on the agar surface, eliminating any potential solvent interference. The disks were subsequently placed onto the surface of inoculated agar plates, which were then held at 4 °C for 2 h to allow adequate pre-diffusion of pyocyanin into the agar matrix before transfer to 37 °C for incubation. Gentamicin (10 µg) (Bioanalyse, Ankara, Turkey) and fluconazole (25 µg) (HiMedia, Mumbai, India) served as positive controls for bacteria and yeasts, respectively. Plates were incubated at 37 °C for 24 h under aerobic conditions. Antimicrobial activity was quantified by measuring the inhibition zone diameter (mm) around each disk. All experiments were performed in triplicate (*n* = 3).

### 3.6. Statistical Analysis

Experimental data for pyocyanin production optimization were analyzed using Design-Expert^®^ software (Version 13, Stat-Ease Inc., Minneapolis, MN, USA). Analysis of Variance (ANOVA) was performed to evaluate the statistical significance of the regression model, individual terms, and interaction effects. The significance of each coefficient was determined using F-test and *p*-values, with a 95% confidence level (α = 0.05). Response surface plots were generated to visualize the interaction effects between variables on pyocyanin production. Antimicrobial activity data were analyzed using IBM SPSS Statistics 20 (IBM Corp., Armonk, NY, USA). A one-way ANOVA was performed to determine significant differences between pyocyanin concentrations for each microorganism. Tukey’s Honestly Significant Difference (HSD) post hoc test was applied for pairwise comparisons when ANOVA indicated significant differences (*p* < 0.05). All data are presented as mean ± standard deviation (SD) from three independent replicates (*n* = 3).

## 4. Conclusions

This study demonstrates that Box–Behnken Design-based response surface methodology provides a statistically robust and experimentally efficient framework for optimizing pyocyanin production by *P. aeruginosa* OG1. The second-order polynomial model exhibited exceptional predictive accuracy (R^2^ = 0.9969, F = 176.3, *p* < 0.0001), and the optimized fermentation conditions—glycerol at 1.11% (*w*/*v*), peptone at 17.86 g/L, and a pH of 7.27—yielded a maximum predicted pyocyanin concentration of 25.41 mg/L. The dominance of quadratic terms in the model underscores the nonlinear nature of the relationships between medium components and pigment biosynthesis, with the significant glycerol × pH interaction highlighting the necessity of simultaneous, rather than independent, optimization of these parameters. Antimicrobial evaluation revealed that pyocyanin exerted dose-dependent inhibitory activity against all seven tested pathogens. The highest efficacy was recorded against *B. cereus* (26.4 ± 1.3 mm at 40 µg/mL), a finding consistent with the known differential susceptibility of Gram-positive bacteria to phenazine-based compounds. In particular, the antifungal activity of pyocyanin against *C. glabrata* (21.5 ± 1.6 mm) was similar to that of the fluconazole-positive control (20.7 ± 1.4 mm), suggesting that it could be considered an alternative antifungal agent against azole-resistant *Candida* strains. The limited but detectable activity against MRSA *S. aureus* (9.2 ± 1.1 mm) warrants further investigation into combination strategies and mechanistic characterization of strain-specific resistance. Collectively, these findings establish BBD-based RSM as a powerful tool for scalable microbial pigment production and position pyocyanin as a promising naturally derived antimicrobial candidate. Future studies should prioritize MIC and MBC determination, cytotoxicity profiling, and mechanistic characterization to establish a more complete antimicrobial profile of pyocyanin. Subsequently, in vivo validation and pilot-scale fermentation optimization would be warranted to assess its broader applicability in pharmaceutical and biotechnological contexts.

## Figures and Tables

**Table 1:** RSM experimental design for the optimization of glycerol, peptone, and pH in pyocyanin production by *P. aeruginosa*.

Run No.	Glycerol (A) (% *w*/*v*)	Peptone (B) (g/L)	pH (C)	Pyocyanin (mg/L)
1	0.5	8	7	6.4
2	1.5	8	7	10.7
3	0.5	24	7	9.9
4	1.5	24	7	16.5
5	0.5	16	6	8.3
6	1.5	16	6	12.9
7	0.5	16	8	11.1
8	1.5	16	8	18.5
9	1.0	8	6	9.1
10	1.0	24	6	14.0
11	1.0	8	8	13.0
12	1.0	24	8	20.0
13	1.0	16	7	24.7
14	1.0	16	7	24.6
15	1.0	16	7	23.9

**Table 2:** ANOVA analysis results for pyocyanin yield.

Source	DF	Adj SS	Adj MS	F-Value	*p*-Value
Model	9	525.95	58.44	176.3	<0.0001
Linear	3	316.48	105.49	318.3	<0.0001
Glycerol	1	101.02	101.02	304.7	<0.0001
Peptone	1	124.8	124.8	376.5	<0.0001
pH	1	90.66	90.66	273.4	<0.0001
Square	3	199.05	66.35	200.1	<0.0001
Glycerol×Glycerol	1	65.61	65.61	197.9	<0.0001
Peptone×Peptone	1	73.44	73.44	221.5	<0.0001
pH×pH	1	60	60	181	<0.0001
2-Way Interaction	3	10.42	3.47	10.46	0.013
Glycerol×Peptone	1	4.21	4.21	12.69	0.016
Glycerol×pH	1	2.89	2.89	8.72	0.031
Peptone×pH	1	3.32	3.32	10.02	0.024
Error	5	1.66	0.33		
Lack of Fit	3	1.28	0.43	2.24	0.33
Pure Error	2	0.38	0.19		
Total	14	527.61			

**Table 3:** Coded coefficients of the regression model.

Term	Coef	SE Coef	T-Value
Constant	24.400	0.257	94.94
Glycerol	3.550	0.183	19.40
Peptone	3.950	0.183	21.58
pH	2.750	0.183	15.02
Glycerol×Glycerol	−4.050	0.273	−14.84
Peptone×Peptone	−4.450	0.273	−16.31
pH×pH	−3.850	0.273	−14.10
Glycerol×Peptone	0.650	0.260	2.50
Glycerol×pH	0.450	0.260	1.73
Peptone×pH	0.550	0.260	2.12

**Table 4:** Antimicrobial activity of pyocyanin against selected bacterial and fungal strains determined by disk diffusion assay (mm ± SD).

Microorganism	Pyocyanin (20 µg/mL)	Pyocyanin (30 µg/mL)	Pyocyanin (40 µg/mL)	Gentamicin (10 µg)	Fluconazole (25 µg)
*Bacillus cereus*	18.3 ± 1.2 ^a^	22.1 ± 1.5 ^b^	26.4 ± 1.3 ^c^	28.6 ± 1.4 ^c^	—
*Candida glabrata*	11.2 ± 1.1 ^a^	16.8 ± 1.3 ^b^	21.5 ± 1.6 ^c^	—	20.7 ± 1.4 ^c^
*Klebsiella pneumoniae*	12.4 ± 1.0 ^a^	15.3 ± 1.2 ^b^	17.6 ± 1.4 ^c^	22.9 ± 1.3 ^d^	—
*Candida albicans*	9.8 ± 0.9 ^a^	12.6 ± 1.5 ^a^	15.4 ± 1.8 ^b^	—	21.3 ± 1.6 ^c^
*Candida parapsilosis*	8.9 ± 1.0 ^a^	10.7 ± 1.3 ^a^	13.2 ± 1.9 ^b^	—	19.8 ± 1.5 ^c^
*Proteus mirabilis*	7.6 ± 0.8 ^a^	10.8 ± 1.1 ^b^	12.5 ± 1.3 ^b^	24.8 ± 1.2 ^c^	—
*Staphylococcus aureus* (MRSA)	6.4 ± 0.7 ^a^	7.9 ± 0.9 ^a^	9.2 ± 1.1 ^a^	0.0 ^b^	—

Values are expressed as mean ± standard deviation (mm inhibition zone, *n* = 3). Different superscript letters within the same row indicate statistically significant differences (one-way ANOVA followed by Tukey’s HSD test, *p* < 0.05). “—“ indicates not applicable. MRSA: methicillin-resistant Staphylococcus aureus.

## Data Availability

The original contributions presented in this study are included in the article. Further inquiries can be directed to the corresponding author.
